# Place Aux Enfants

**Published:** 1918-08-10

**Authors:** 


### SOME PROBLEMS OF TO-DAY.

### Place aux Enfants.

### By A SOCIAL WORKER.

CHILDREN, we are continually reminded, are the most precious asset of the nation. Where does the nation keep these living jewels, for whose better polishing so much thought is at this moment being taken? Chiefly, we must answer, in the streets of its cities, and that without over-careful choice of those which are cleanest, airiest, and most free from traffic. Where is the infant, where is the toddler, to have its being during those working hours of the mother which are also the elder children's schoolhours? Once the cradle stage is passed the choice is practically between the dusty and draughty floor of kitchen or living-room and the dusty pavement outside, and the latter offers the fresher air. Babies of the class where pretensions to genteelness begin have often paler faces than those on a lower social level because they are kept indoors till the mother can find time to dress them adequately and wheel them out for air. If she is not one of the "*most* perticikler" about appearances she may anchor the "pram" in front of her door, or if more anxious about appearances than doubtful of the influences of sink-drains and dust-bins she may put it out in her tiny back yard. The toddler, however, cannot be left in the pram unless asleep. Out of schoolhours older children can be detailed to nurse the infant and chaperon the toddler out of doors, and the rest of the children will naturaly be sent to play there, at once within call and out of the small rooms which have enough to do to find floor-space for the necessary furniture.

FRESH AIR AND PLAY IN SAFE CONDITIONS.

Mothers are not indifferent to the drawbacks of the street. They constantly send the family party to any park or open space within reach in charge of the elder children, who have strict instructions to inquire the *right* time—an injunction carefully followed, to the frequent annoyance of watch-owners—and to return by a given hour. Tube stations in certain neighbourhoods serve an invaluable purpose in winter as crèches. As regards the older children, mothers have lately been on the horns of two different dilemmas. They are being scolded on the one hand for an increase in juvenile crime as the result of uncontrolled leisure, on the other for the detriment to children's health resulting from paid work done out of school hours. Both facts are patent and are regrettable. Certain clauses in the new Education Bill prove the national conviction that "something must be done."

HOW TO LESSEN JUVENILE CRIME.

Meantime the existence of child-labour and especially boy-labour is unfairly supposed to be due to a selfish parental desire to exploit the child for gain. There are bad cases of this, but far more often the object has been to divert energy into safe channels. The organised games of term-time, the country sports and boating arranged for boys of a higher class, are understood to be needed not only or even chiefly as harmless amusement, but as invaluable training. As Mr. Thomas Holmes long ago pointed out, the arrangement of organised matches—say, of football—between boys of different elementary schools or urban districts would do more to lessen juvenile crime, to promote physical health, than any police work in the one case or cheap trips in the other. Before the war there was no more depressing sight in London than that of the serried masses of lads smoking and betting while looking on at inter-club matches. Serious persons had at that time begun to call on the mothers and beseech them to avoid blind-alley occupations, pointing out with truth that a seat on the tailboard of a van from which the boy would be deposed when he came to man's weight and wages led to nothing. It had, however, been a safe and healthy refuge from that unsatisfactory continuation school the street, and did something to "use 'em to" the idea of working, and the satisfaction of earning.

WHAT TO DO WITH THE GIRLS.

The Scout movement's results show how quickly a boy will generally rise to the enjoyment of strenuous effort. The better the boy, the more readily he puts himself to school, and works up subjects such as languages or cookery for his badges. Presented as "school subjects" he would have viewed them very differently. "Call this education? Why, I like it!" announced an amazed young soldier lately. And as to the girl: childhood past, the careful mother does not want her to learn rough language and rougher manners in the street. Yet her own tenderness, and public opinion, to say nothing of the child's own desire to play, forbid her to put much of her own drudgery on the young shoulders. All she wants is to keep her girl happy and healthy and in safe surroundings and to be free to toil herself. Indeed, the snubbing of a child's willing helpfulness is certainly a common fault of such mothers. It is a pity that our towns have no industry like the lace-making which in Flemish cities collects the feminine element from grandmother to little girl in happy chatting groups seated outside the house-doors. The high steps of our streets would be a pleasant sight if the women sat there sewing and knitting on summer afternoons amongst the children. Too much of the day of a thrifty woman is spent indoors. But—and there is the point to which these reflections lead—what is urgently needed is a co-operating of housing authorities with those responsible for education.

A SUGGESTION TO HOUSING AUTHORITIES

From the great thoroughfares rows of streets commonly lead away to right and left. The yard or garden of each meets the garden of another house, its front door is in the next street. And new districts are rapidly built everywhere on the same principle. But suppose that instead of this gridiron design the houses were arranged in a hollow square, each still keeping a tiny yard but the central space gravelled or asphalted, with some erection for shade, or possibly, as Miss Macmillan suggests, a marquee nursery school in the centre? It cannot be beyond the wit of man—and woman—to devise for the children of the nation a clean, dry, safe place of being, close to the home but yet in the outdoor air. Larger recreation grounds where games can be played, furnished also with swimming baths, will become general, no doubt as a result of "continued education." But it is not difficult to imagine that a few centuries hence our descendants will look at some old town maps and ask in perplexity:

"But where, then, did they keep the children?"

